# Retrospective Analysis of Chilean and Mexican GI Stromal Tumor Registries: A Tale of Two Latin American Realities

**DOI:** 10.1200/JGO.19.00410

**Published:** 2020-04-23

**Authors:** Germán Calderillo, Matías Muñoz-Medel, Edelmira Carbajal, Miguel Córdova-Delgado, Doris Durán, Ignacio N. Retamal, Piga Fernández, Absalón Espinoza, Rodrigo Salas, María de la Paz Mastretta, Héctor Galindo, Bruno Nervi, Jorge Madrid, Cesar Sánchez, Carolina Ibáñez, José Peña, Sebastián Mondaca, Francisco Acevedo, Erica Koch, Mauricio P. Pinto, Marcelo Garrido

**Affiliations:** ^1^ Gastroenterology Oncology Chief Division, National Cancer Institute, México City, México; ^2^ Department of Hematology and Oncology, School of Medicine, Pontificia Universidad Católica de Chile, Santiago, Chile; ^3^ Fundación GIST México, San Pedro Garza García, México; ^4^ Faculty of Medicine and Science, Universidad San Sebastián, Santiago, Chile; ^5^ Faculty of Dentistry, Universidad de los Andes, Santiago, Chile; ^6^ Fundación GIST Chile, Santiago, Chile; ^7^ Instituto Médico del Seguro Social–Unidad Médica de Alta Especialidad No. 25, Monterrey, México

## Abstract

**PURPOSE:**

Like other malignancies, GI stromal tumors (GIST) are highly heterogeneous. This not only applies to histologic features and malignant potential, but also to geographic incidence rates. Several studies have reported GIST incidence and prevalence in Europe and North America. In contrast, GIST incidence rates in South America are largely unknown, and only a few studies have reported GIST prevalence in Latin America.

**PATIENTS AND METHODS:**

Our study was part of a collaborative effort between Chile and Mexico, called Salud con Datos. We sought to determine GIST prevalence and patients’ clinical characteristics, including survival rates, through retrospective analysis.

**RESULTS:**

Overall, 624 patients were included in our study. Our results found significant differences between Mexican and Chilean registries, such as stage at diagnosis, primary tumor location, CD117-positive immunohistochemistry status, mitotic index, and tumor size. Overall survival (OS) times for Chilean and Mexican patients with GIST were 134 and 156 months, respectively. No statistically significant differences in OS were detected by sex, age, stage at diagnosis, or recurrence status in both cohorts. As expected, patients categorized as being at high risk of recurrence displayed a trend toward poorer progression-free survival in both registries.

**CONCLUSION:**

To the best of our knowledge, this is the largest report from Latin America assessing the prevalence, clinical characteristics, postsurgery risk of recurrence, and outcomes of patients with GIST. Our data confirm surgery as the standard treatment of localized disease and confirm a poorer prognosis in patients with regional or distant disease. Finally, observed differences between registries could be a result of registration bias.

## INTRODUCTION

GI stromal tumors (GISTs) are relatively rare mesenchymal tumors that arise from GI tract walls.^1^ Studies have postulated that GISTs are generated in a specific subset of cells called interstitial cells of Cajal.^2^ These are pacemaker cells that form a complex cellular network that coordinates peristaltic movements, and these cells express both neural and myoid features. As occurs with other malignancies, GISTs are highly heterogeneous. This applies not only to tumor histologic features and malignant potential, but also to incidence rates. Indeed, studies demonstrate that GIST morphology is highly variable, the presentation of GIST varies from virtually benign to highly aggressive, and incidence rates range from 4.3 to 22.0 per million in different geographic areas.^3^ In addition, the incidence of GIST is similar in males and females and GIST can occur at any age; however, the reported median age at diagnosis is usually between age 50 and 70 years. Most cases are sporadic^4^ and stain positive for KIT (also called CD117^5^), a tyrosine protein kinase considered the main oncogenic driver of GISTs.

Complete surgical resection remains the standard treatment of patients with localized GIST. In addition, some patients receive preoperative and/or adjuvant therapy with imatinib, a tyrosine kinase inhibitor (TKI) that targets KIT.^1^ For patients with advanced GIST (either regional or distant disease), chemotherapy and radiotherapy are largely ineffective,^6^ and standard treatment may include a variety of TKIs, such as imatinib, sunitinib, or regorafenib.^1^

As mentioned earlier, GIST incidence across different geographic areas is highly heterogeneous. In Latin America, a single systematic review in Mexico reported an incidence of 9.7 cases per million population.^7^ In South America, GIST incidence is largely unknown, and only a single study in Peru has reported a GIST registry that includes 103 patients and their basic characteristics.^8^ Here, we report the results of a collaborative initiative between Chile and Mexico called Salud con Datos, which includes a total of 624 patients with GIST. To the best of our knowledge, this is the largest study on GIST within Latin America. Overall, our data indicate significant differences between registries from Chile and Mexico, confirming the heterogeneity of the disease. We also discuss potential registration bias and similarities and differences that may reflect different realities between our 2 countries.

**CONTEXT**

**Key Objective**
To compare two Latin American GI stromal tumor registries, evaluating their similarities and differences in prevalence, demographics and survival.
**Knowledge Generated**
Surgery is confirmed as the gold standard treatment of localized disease. Also, an unexpected high heterogeneity in the proportion of localized, regional, and distant disease is observed between Latin American countries.
**Relevance**
This is the largest report on the prevalence of GI stromal tumors in Latin America involving two countries, Chile and Mexico.

## PATIENTS AND METHODS

### Participating Institutions

This study included clinical data from a total of 624 patients with GIST; 521 patients were contributed by the Fundación GIST Mexico and 103 by Fundación GIST Chile. This joint work is the result of the Salud con Datos initiative, which was initially formed by both advocacy groups as part of Alianza GIST, a Latin American nonprofit organization focused on the development of research, public policies, and improved access to specific treatments for Latin American patients with GIST. The registry was funded by the Life Raft Group, an international, nonprofit patient advocacy organization created to enhance survival and quality of life for people living with GIST through patient-powered research, education, and empowerment and global advocacy efforts. Life Raft Group had no access to patient raw data and did not participate in the database setup, data acquisition, or analysis during this study. This study was designed as an observational, multicenter, retrospective registry and was approved by the institutional review board and ethics committee of all participating institutions, following the Declaration of Helsinki, Good Clinical Practices, and both Mexican and Chilean regulations. Written informed consent was obtained from all participating patients.

### Data Acquisition

Patients were enrolled from August 2016 through February 2019. Chilean data were collected from medical records at participating institutions, and Mexican data were obtained from patient registry information at Fundación GIST Mexico. The database included basic information, demographic characteristics, onset symptoms, tumor characteristics, diagnostic procedures, treatment regimen (if any), and clinical outcomes. All designated data entry personnel received prior training and were responsible for entering data into the registry. To assess the quality of the data, a trained monitor for the study periodically visited each participating center to review the relevant patient medical records.

### Inclusion and Exclusion Criteria

The registry included individuals of all ages diagnosed with histologically confirmed GISTs who had at least 3 months of follow-up with access to clinical information. Patients were excluded if they had missing or incomplete information, had missing clinical follow-up data, or were unable or unwilling to sign written informed consent.

### Statistical Analysis

Continuous variables entered in the registry were expressed as mean ± standard deviation values or as median and range (minimum and maximum) values according to their distribution (normal *v* not normal). Categorical variables were expressed as percentages. Statistical comparisons among groups were performed using the *t* test when data were normally distributed; otherwise, the Mann-Whitney *U* test was performed. The distribution of continuous variables for more than 2 groups was analyzed using analysis of variance or the Kruskal-Wallis test, depending on data normality. Categorical variables were expressed as percentages. The differences in categorical variables were determined using Fisher’s exact test.

### Survival Curve Analysis

Overall survival (OS) was defined as time from diagnosis of GIST to death from any cause. Progression-free survival (PFS) was defined as time from diagnosis of GIST to disease progression. Survival curves were calculated using the Kaplan-Meier method, and different variables were compared using the log-rank test. All statistical analyses were performed using R statistical software. All analyses were 2-tailed, and significance was set at *P* ≤ .05.

### Assessment of GIST Recurrence Risk

We used two assessments to calculate recurrence risk, the modified National Institutes of Health (NIH) criteria and Armed Forces Institute of Pathology (AFIP) criteria. The modified NIH criteria use two clinical pathologic factors, such as tumor size and mitotic count. The recurrence risk is stratified as very low, low, intermediate, or high. The AFIP criteria use tumor site, tumor size, and mitotic count. Tumor size is categorized into the following four groups: < 2 cm, > 2 to ≤ 5 cm, > 5 to ≤ 10 cm, and > 10 cm. The mitotic count is classified into the following two groups: ≤ 5 or > 5 mitoses per 50 high-power fields. Tumor sites identified in the classification are stomach, duodenum, ileum/jejunum, and rectum.^9^

## RESULTS

### Patient Characteristics and Demographic Information

A total of 624 patients with GIST were recruited in Chile and Mexico as part of the Salud con Datos collaborative initiative. In Chile, 103 patients were recruited at 40 institutions, whereas in Mexico, a total of 521 patients were recruited at 79 centers, referred by Fundación GIST Chile and Fundación GIST Mexico, respectively. General demographic and histopathologic characteristics are listed in Table 1. Median age at diagnosis was not statistically significantly different between the registries (54 and 53 years in Chile or Mexico, respectively; *P* = .38). However, other measured characteristics, including stage at diagnosis, primary tumor location, CD117-positive immunohistochemistry status, mitotic index, and tumor size, demonstrated significant differences between the two registries. Consequently, most data are presented separately for Chile and Mexico. Despite these differences, in both registries, stomach was the most frequent tumor site (53.4% and 46.6% for Mexico and Chile, respectively). In addition, both registries showed internal consistency when comparing male and female basic characteristics (Appendix Table A1).

**TABLE 1:**
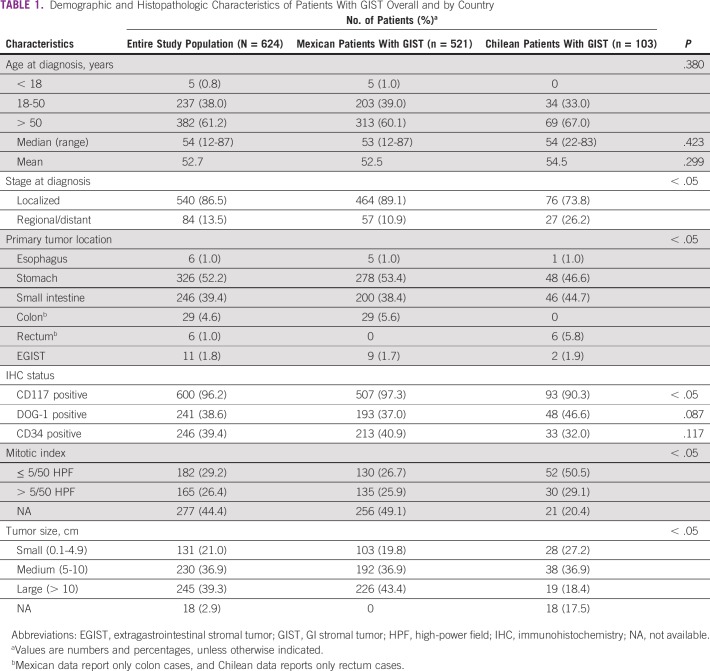
Demographic and Histopathologic Characteristics of Patients With GIST Overall and by Country

### Survival Rates in the Chilean and Mexican Registries

**FIG 1:**
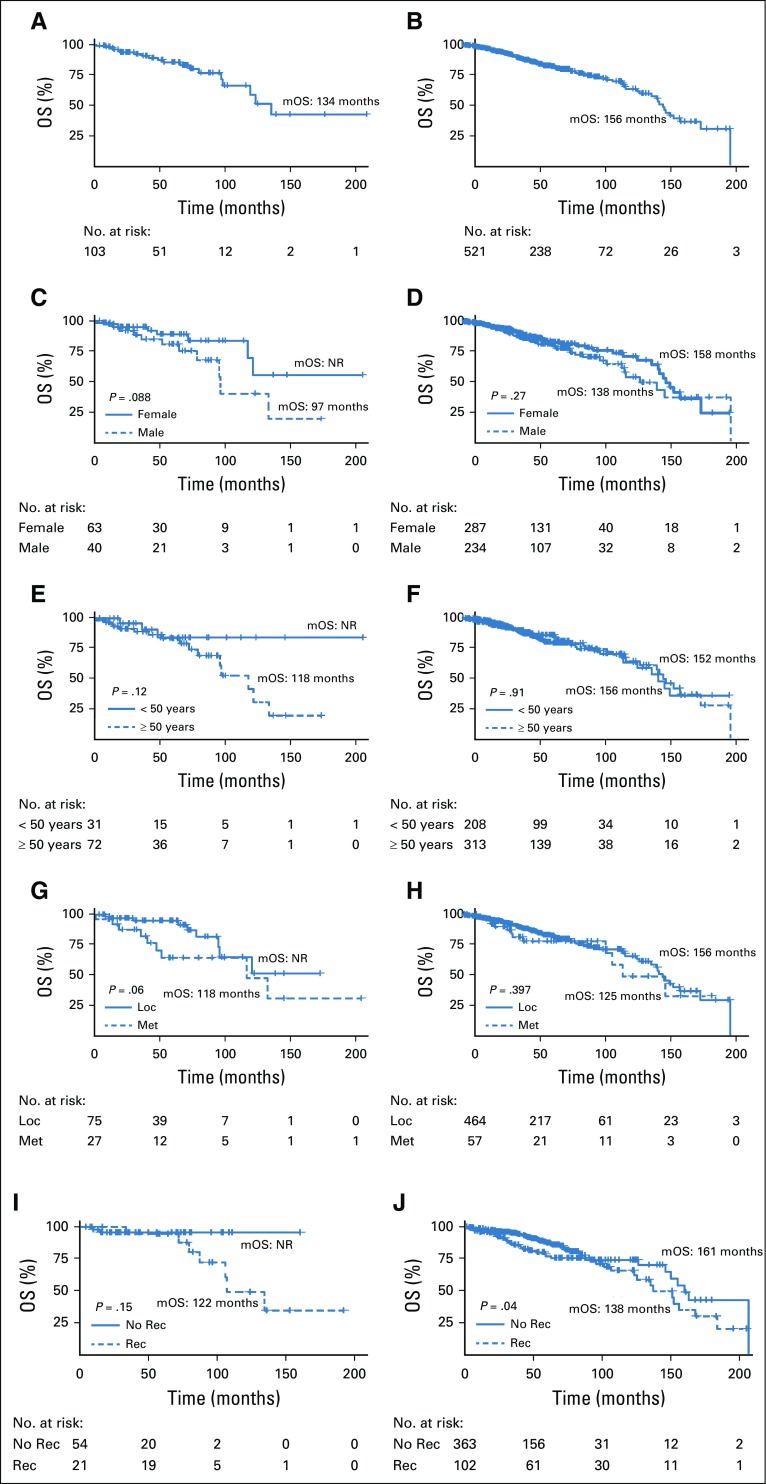
Overall survival (OS) Kaplan-Meier curves in Chilean (n = 103) and Mexican (n = 521) patients with GI stromal tumors (GISTs). (A) Chilean patients; (B) Mexican patients; (C) Chilean patients with GIST by sex; (D) Mexican patients with GIST by sex; (E) Chilean patients with GIST stratified by age at diagnosis; (F) Mexican patients with GIST stratified by age at diagnosis; (G) Chilean patients with GIST by stage at diagnosis; (H) Mexican patients with GIST by stage at diagnosis; (I) Chilean patients with localized (Loc) GIST by recurrence status during follow-up; and (J) Mexican patients with localized GIST by recurrence status during follow-up. Met, metastatic; mOS, median overall survival; No Rec, no recurrence; NR, not reached; Rec, recurrence.

**FIG 2:**
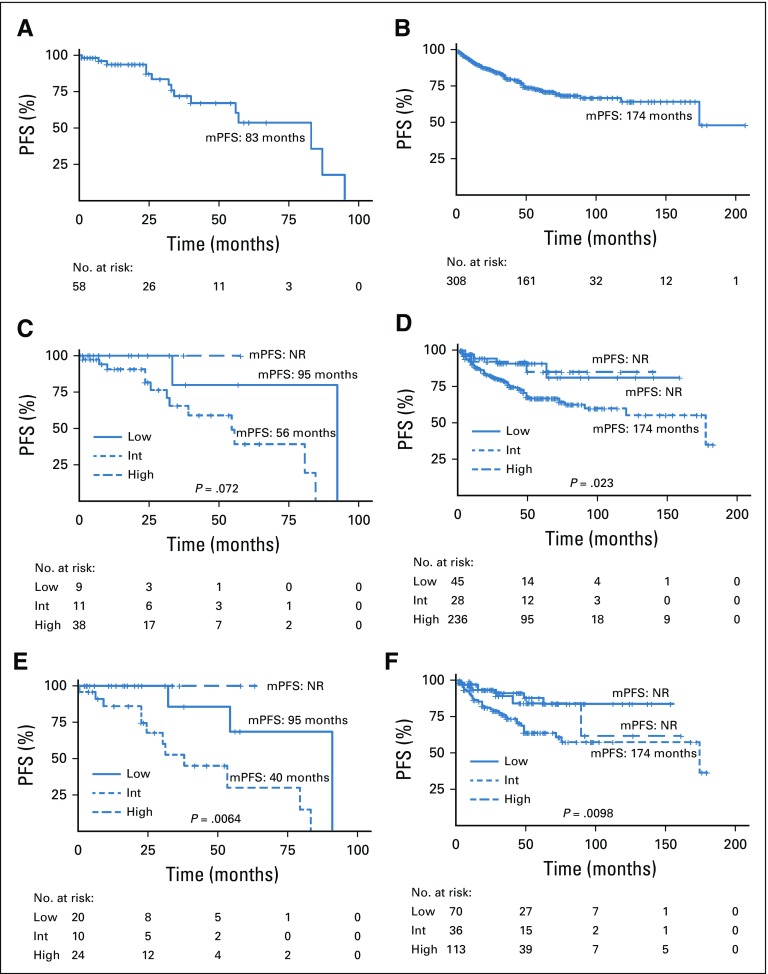
Progression-free survival (PFS) Kaplan-Meier curves in patients with localized, resected GI stromal tumors (GISTs). (A) Chilean patients (n = 58); (B) Mexican patients (n = 309); (C) Chilean patients stratified by modified National Institutes of Health (NIH) risk criteria (n = 68); (D) Mexican patients stratified by modified NIH risk criteria (n = 309); (E) Chilean patients with localized GIST stratified by Armed Forces Institute of Pathology (AFIP) risk system (n = 63); and (F) Mexican patients with localized GIST stratified by AFIP risk system (n = 226). Int, intermediate; mPFS, median progression-free survival; NR not reached.

We evaluated OS (Fig 1) and PFS (Fig 2) rates comparing the Chilean and Mexican registries (Appendix Table A2). Figures 1A and 1B show OS rates for the entire Chilean and Mexican cohorts (median OS, 134 and 156 months, respectively).

Figure 1C shows OS rates analyzed by sex; in the Chilean cohort, females displayed a trend toward better OS that did not reach statistical significance (*P* = .088 by log-rank test; Fig 1C). Figure 1D shows that the median OS times for males and females in the Mexican cohort were 138 and 158 months, respectively (*P* = .27 by log-rank test). Similarly, no statistically significant differences in OS were detected by age (Figs 1E and 1F), stage at diagnosis (Figs 1G and 1H), or recurrence status (within localized disease subset; Figs 1I and 1J) in both cohorts.

### PFS

PFS rates were calculated in both registries. Median PFS times for the entire Chilean and Mexican cohorts were 83 and 174 months, respectively (Figs 2A and 2B). We then assessed PFS by the two risk of recurrence stratification methods—the modified NIH and AFIP criteria. Only patients with the assessment criteria were included in this analysis.

Using the first assessment system (modified NIH criteria), the high-risk group in the Chilean cohort displayed a trend toward a poorer PFS compared with the intermediate- and low-risk groups; however, these differences did not reach statistical significance (*P* = .072 by log-rank test; Fig 2C). In contrast, the low-risk group in the Mexican cohort had a significantly better PFS (*P* = .023; Fig 2D). Finally, using the AFIP criteria to assess risk of recurrence, we found significant differences among the three risk subgroups in both the Chile (*P* = .0064 by log-rank test; Fig 2E) and Mexico cohorts (*P* = .0098 by log-rank test; Fig 2F).

## DISCUSSION

To the best of our knowledge, this is the largest report on the prevalence of GIST in Latin America and involves two countries (Chile and Mexico) and a total of 119 health institutions. Worldwide, the largest study on GIST includes > 6,000 patients obtained from the SEER database in the United States.^10^ This study demonstrated an increase in incidence of GIST from 5.5 per million in 2001 to 7.8 per million in 2011. A previous Latin American study included a systematic review that comprised a total of 11 published articles in the Mexican population^7^; this study provided a GIST incidence estimate of 9.7 cases per million, which is based on data accrued over a decade. In addition, a couple of GIST registries from Peru^8^ and Mexico^11^ have reported 103 and 275 GISTs, respectively.

As mentioned earlier, our data from Chile and Mexico were analyzed separately; average and median ages at diagnosis were 54.5 and 54 years in Chile and 52.5 and 53 years in Mexico. Interestingly, patients in our study were diagnosed at a younger age compared with previous reports that have indicated a range between 58 and 61 years at diagnosis.^10-15^ In addition, the Peruvian GIST registry reports an average age of 64 years at diagnosis.^8^ As occurs with GIST incidence in the United States, we speculate this difference could be attributed to better screening, disease awareness, and application of GIST histology codes over time.^10^ Regarding the proportion of males and females, our data from Chile indicate a predominance of female patients (61.2%). Similarly, data from Mexico also indicated a slight predominance of female patients (55.1%). Previous reports on this matter are inconsistent; most European studies describe a similar proportion of both sexes, in some cases with a slight predominance of female patients.^16-19^ However, studies from the United States^10^ and Nordic countries^20,21^ demonstrate a predominance of male patients. In terms of staging, GISTs can be classified as localized, regional, or distant. In general, studies indicate a higher proportion of localized GISTs versus regional or distant GISTs; however, these proportions can vary wildly among studies. For example, the SEER database reports that 54.4% of GISTs in the United States are localized.^10^ In sharp contrast, a French report indicates a 91.6% rate of localized GISTs.^19^ Accordingly, our data demonstrate a significant difference in the percentage of localized GISTs between the Mexican registry (89.1%) and Chilean registry (73.8%). We speculate that this is a registration bias in our study, explained by the medical staff that developed the registry. In Mexico, the registry involves a series of health institutions and a staff led by surgeons. In contrast, the Chilean registry was developed by a group led by medical oncologists. Consequently, the Chilean registry contains a larger proportion of advanced GISTs. This difference might also explain the lower OS rates observed in the Chilean cohort (Figs 1A and 1B). Evidently, this registration bias is a limitation of our study. As expected, the most frequent site of primary tumors in our study was the stomach (46.6% and 53.4% for Chile and Mexico, respectively). Similarly, the majority of patients (96.2% overall) were CD117 positive, in line with reports that indicate a 95% rate of CD117 positivity.^5^

The malignant potential of GISTs is highly heterogenous, ranging from virtually benign to aggressive, rapidly progressing tumors. In this regard, an estimation of the risk of recurrence in patients with localized disease may be relevant to decide potential adjuvant treatments after surgery. A variety of validated stratifications systems can accurately assess the risk of recurrence, and these methods are largely equivalent.^1,22^ Our study assessed risk of recurrence using the modified NIH^23^ and AFIP^24^ criteria. These systems are based on the following tumor characteristics: size (diameter), mitotic counts, site, and rupture. As expected, high-risk patients displayed shorter PFS with both systems, except for the modified NIH criteria in the Chilean cohort (Fig 2C), which displayed a trend that did not reach statistical significance (*P* = .072). Unexpectedly, patients with low risk of recurrence in the Chilean cohort displayed shorter PFS compared with patients categorized as intermediate risk (Figs 2C and 2E). We speculate this discrepancy could be the result of an inaccurate assessment of mitotic counts that overestimated or underestimated the risk of recurrence; in addition, these inaccuracies are more evident given the low number of patients in these subsets (n = 12 and n = 11 with low and intermediate risk, respectively; Table 2). Indeed, a portion of these patients received preoperative imatinib, which may have affected mitotic counts, underestimating the risk of recurrence. A similar phenomenon affected the Mexican cohort. Looking forward, these observations and risk assessments should be recalculated and evaluated in a larger sample.

**TABLE 2:**
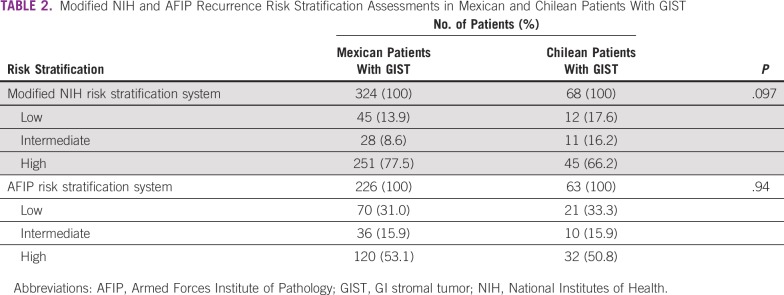
Modified NIH and AFIP Recurrence Risk Stratification Assessments in Mexican and Chilean Patients With GIST

To our knowledge, we report the largest study on GISTs in Latin America. Our data confirm surgery as the gold standard treatment of localized disease. Our study also confirms previous reports that indicate a high heterogeneity in the proportion of localized, regional, and distant disease. Indeed, data from registries in Chile and Mexico display significant differences, probably as a result of registration bias. These registries represent the following two realities: a team led by medical oncologists who preferentially enrolled patients with regional or distant GIST and a large multicenter registry led by surgeons who enrolled mostly patients with localized GIST. Evidently, differences in OS and PFS between these cohorts can be attributed to these differences in enrollment.

Our study has a number of limitations. First, as mentioned earlier, registration bias may limit the scope of our findings. Second, data from both registries were accrued retrospectively. Third, mitotic counts were not available for a large subset of patients with localized GIST, especially in the Mexican cohort. Therefore, the assessments of the risk of recurrence were not available for all enrolled patients. Finally, a subset of patients categorized as being at low risk of recurrence received preoperative imatinib, which may have altered mitotic counts. Therefore, the risk assessment in these patients could not be estimated reliably.

Despite the differences between these Chile and Mexico, a collaborative effort of this magnitude is a contribution to the characterization of GIST. Salud con Datos aims to maintain, grow, and improve these alliances and their results in the future.

## Figures and Tables

**TABLE A1:**
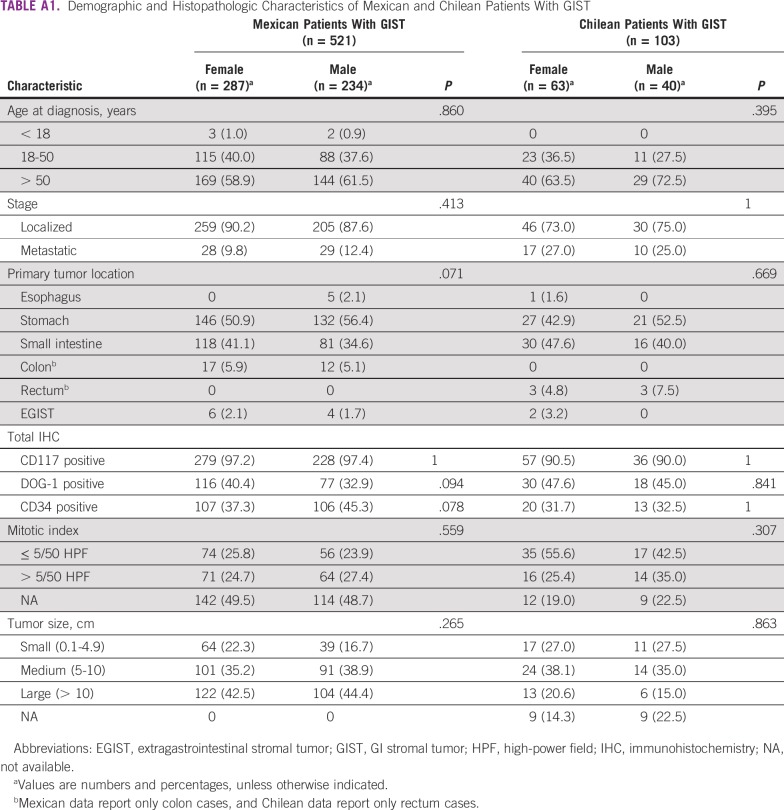
Demographic and Histopathologic Characteristics of Mexican and Chilean Patients With GIST

**TABLE A2:**
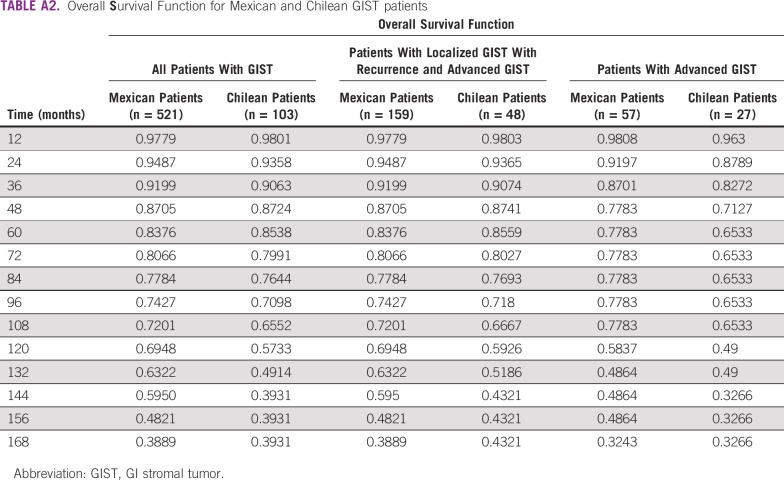
Overall **S**urvival Function for Mexican and Chilean GIST patients
